# RD-1 encoded EspJ protein gets phosphorylated prior to affect the growth and intracellular survival of mycobacteria

**DOI:** 10.1038/srep12717

**Published:** 2015-07-31

**Authors:** Pramod K Singh, Richa Saxena, Sameer Tiwari, Diwakar K Singh, Susmita K Singh, Ruma Kumari, Kishore K Srivastava

**Affiliations:** 1 Division of Microbiology and Academy of Scientific and Innovative Research, CSIR-Central Drug Research Institute, Lucknow, 226031, India

## Abstract

*Mycobacterium tuberculosis* (MTB) synchronizes a number of processes and controls a series of events to subvert host defense mechanisms for the sake of residing inside macrophages. Besides these, MTB also possesses a wide range of signal enzyme systems, including eleven serine threonine protein kinases (STPKs). The present study describes STPK modulated modification in one of the hypothetical proteins of the RD1 region; EspJ (ESX-1 secretion associated protein), which is predicted to be involved in virulence of MTB. We have employed knock-out MTB, and *M. bovis* BCG as a surrogate strain to elaborate the consequence of the phosphorylation of EspJ. The molecular and mass spectrometric analyses in this study, confirmed EspJ as one of the substrates of STPKs. The ectopic expression of phosphoablative mutants of *espJ* in *M. bovis* BCG also articulated the effect of phosphorylation on the growth and in survival of mycobacteria. Importantly, the level of phosphorylation of EspJ also differed between pathogenic H_37_ Rv (Rv) and non pathogenic H_37_ Ra (Ra) strains of MTB. This further suggested that to a certain extent, the STPKs mediated phosphorylation may be accountable, in determining the growth and in intra-cellular survival of mycobacteria.

Characteristically, the phosphorylation engages a wide range of proteins in mycobacteria and most of them regulate cell wall biosynthesis. A number of findings have suggested that phosphorylation in the proteins which are associated with the virulence factors affect the mycobacterial physiology^1^. As an example, phosphorylation of VirS by PknK controls the *mymA* operon (Rv3083–Rv3089) which is involved in the biosynthesis of mycolic acids, presumably via a FAS-II-independent pathway. This is important for maintaining cell wall integrity^2^, and later increases its affinity for the *mym* promoter DNA^3^. Similarly, PknA and PknB phosphorylate several proteins, including Wag31 (Rv2145c)^4^, a homolog of the cell shape/cell division protein DivIVA which is essential for mycobacterial growth^5^. The role of PknA in controlling cell division was also confirmed in studies demonstrating a direct interaction between PknA and MTB FstZ; a protein central to the bacterial septum formation. The Penicillin-binding proteins (PBPs) of mycobacteria are the other examples which participate in the cell wall expansion, cell shape maintenance, septum formation and in cell division. One of these proteins, PbpA (Rv0016c), is also reported to be phosphorylated by PknB^6^.

Besides STPKs; signaling in mycobacteria is also controlled by two component system. The putative transcription regulator factors PhoP/PhoR, in MTB, are the part of this system which control transcription of key virulence genes essential for survival^7^. Mechanism behind attenuation of Ra strain has also been correlated with differential amount of secretion of RD1 encoded protein ESAT-6 in Rv and in Ra strains which might be regulated by PhoP^8^. Nevertheless, molecular mechanisms behind differential activity of these proteins are still unknown.

Expressions of STPKs as proteins involved in pathogenesis, have also been reported earlier^9^, suggesting differential control of signaling in diverse strains. Although, the genetic rationale for diminished virulence of MTB Ra has been elucidated^10^ to reveal the comparative behavior, but the molecular mechanism is still unidentified. Nevertheless RD1 region as well as all the STPKs are co-inherited in both the strains. It is very plausible to infer that co-inheritance of STPKs and RD1 locus in these strains tune the physiology of MTB which modulate their differential behavior (Table 1). A recent study on the comparative gene expression analysis has identified 22 genes which were consistently expressed at higher levels in Rv than in Ra under a variety of growth conditions, and among them seven of the genes were involved in cell wall and cell processes^11^.

We have investigated the interrelationships between EspJ (encoded by Rv3878) with STPKs of mycobacteria and their differential behavior in pathogenic Rv and in non-pathogenic Ra strains. EspJ, so far known as a hypothetical protein, has been putatively categorized as a regulatory protein^12^ and annotated under functional category “cell wall and cell process” in Tuberculist database. This protein is present both in Ra and in Rv strains of mycobacteria but is absent in *M. bovis* BCG. Herein, we have elucidated the role of phosphorylated and un-phosphorylated EspJ in the growth of mycobacteria. Surprisingly, a higher degree of phosphorylation in Rv was observed over Ra which may imply, the distinctive behavior of this protein in pathogenic and in non-pathogenic strains. Further, in order to identify the key residues undergoing phosphorylation, we used LC/MS/MS which are potentially being used for the identification of phosphorylation sites at several instances^13^. Using the proteomics and bioinformatics tools, and coupling with the data received through *in vitro* kinase assay, we have identified phosphorylation sites in EspJ. Generation of phosphoablative mutants by site directed mutagenesis, followed by the transfer of these phosphoablative alleles in *M. bovis* BCG; we have deciphered its role in the growth and in persistence of mycobacteria. This phenotype was also confirmed by knocking-out the gene from MTB and then complementing with wild type and phosphoablative genes.

## Results

### Detection of putative phospho-motifs in RD1 encoded proteins

Web based bioinformatics tools like Kinasephos 2.0, Disphos 1.3, Netphos 2.0 and NetPhosBac1 predicted putative phosphorylation sites in RD1 encoded proteins (Table 2). Based on the comparative scores among these proteins, we have predicted EspJ as a possible substrate of mycobacterial kinase. An added criterion for the elaborative study of this protein has also been the presence of Rxx(S/T) motif, which exists in most of the substrates for STPK, including FtsZ protein, which regulates cell division in mycobacteria^14^. Bioinformatics analysis suggested Ser^70^, Ser^85^ and Thr^144^ as other most probable phosphorylation sites in EspJ protein.

### Expressions of EspJ and PknG proteins in different mycobacterial strains

**Figure 1:**
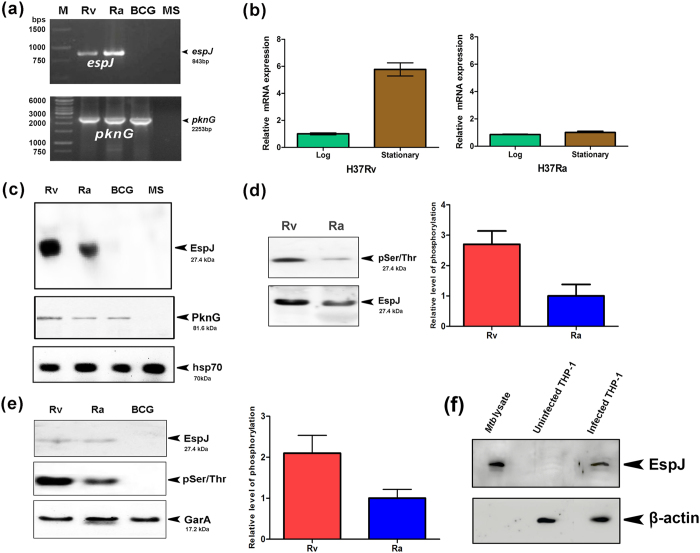
Differential transcription, expression and phosphorylation of EspJ in mycobacteria (**a**) PCR amplification of *espJ* and *pknG* ORF from genomic DNA of Rv, Ra, *M. bovis* BCG (BCG) and *M. smegmatis* MC^2^ 155 (MS). The *pknG* primers specific to MTB did not amplify MS template DNA due to 5’ sequence heterogeneity. Though MS specific primers do amplify MS *pknG* (data not presented) (**b**) Analysis of the *espJ* transcript level in Rv and in Ra strains of MTB. Total RNA was isolated and cDNA was prepared from the different stages of culture of Rv and Ra strains using reverse transcriptase. qRT-PCR was done to analyze the transcript level of *espJ* in cultures of log and stationary phases. Data are shown as means ± SD of triplicate experiments in three biological replicates. (**c**) Immunobloting of mycobacterial (Rv, Ra, BCG and MS) whole cell lysates with EspJ and PknG antisera and hsp70 antibody was performed to show the expression of these proteins in selected lysates. (**d**) Immunoprecipitation of EspJ protein from whole cell lysates of Rv and Ra followed by immunoblotting with anti pSer/Thr antibody to demonstrate differential *in vivo* phosphorylation of EspJ protein. The same blot was re-probed with anti EspJ antiserum to show the level of unphosphorylated protein. Densitometry of the protein bands was done to show the fold change after normalization. (**e**) Detection of phosphorylated EspJ in culture filtrate (CF) of Rv and Ra. Equal amounts of CF proteins harvested from MTB and BCG strains were quantified and resolved by 12% SDS-PAGE. Western blot analysis was done using EspJ antibody. Since, BCG lacks EspJ ORF its CF was used as a negative control. The same blot was re-probed with anti pSer/Thr antibody to demonstrate differential *in vivo* phosphorylation of EspJ protein in Rv and in Ra. Densitometry was done as described. The proteins were immunoblotted with GarA antbody (which is secretory in nature) to show the equal level of loading (lower panel). The blots were also probed with hsp70 antibody to rule out any necrosis during harvesting. Since the bands were undetectable, the data cannot be shown. Besides secretion of EspJ in culture medium, the protein is also found to be present in the cytosol of infected macrophages at 24 h of infection (**f**).

The *espJ* and *pknG* ORFs were amplified by PCR from the genomic DNA obtained from different mycobacterial strains, using gene specific primers. The presence of *espJ* in Rv and Ra, and its absence in BCG and in MS were confirmed by PCR. Likewise, it was observed that the MTB orthologous *pknG* is present only in Rv, Ra and in BCG (Fig. 1a). In order to find out the transcription of *espJ* and to relate with the protein, we quantified the mRNA transcripts of the gene in pathogenic and in non-pathogenic mycobacteria at real time. The results were overwhelming and very conclusive. The nonpathogenic Ra showed a constitutive level of the gene expression in stationary and in log phases of growth with no sign of changes in the transcription level, while pathogenic counterpart Rv has six times higher transcripts in stationary phase as compared to logarithmic phase of growth (Fig. 1b). This suggested that the level of EspJ may play the role in regulating the growth potential in these two strains. Immunoblottings with EspJ and PknG antisera were done to further confirm the expression of EspJ and PknG proteins in different mycobacterial strains (Fig. 1c). Presence of PknG and absence of EspJ in *M. bovis* BCG, enabled us to employ it as a surrogate strain for the present study to address the role of EspJ in mycobacterial physiology.

### Confirmation of EspJ phosphorylation *in vivo* and in culture filtrate proteins of mycobacteria

In order to look for the comparative *in vivo* phosphorylation of EspJ protein in Rv and in Ra, the cell lysates were immunoprecipitated with the EspJ antiserum and then detected by western blotting. An interesting observation was established; showing that the amount of phosphorylated protein in Rv is substantially higher than the Ra (Fig. 1d). Further to affirm the differential phosphorylation of secreted EspJ protein; the log phase culture filtrates of Rv, Ra and BCG, were immunoblotted with EspJ antiserum and with anti-phospho Ser/Thr antibody (Fig. 1e). We questioned that whether the secretory nature of this protein mimics during intracellular infection, the cell lysates of infected and uninfected macrophages were subjected for immunoblotting with anti-EspJ serum. The data confirmed that protein is secreted out in the cytosol of macrophages (Fig. 1f). Although, till at this stage data revealed that EspJ is phosphorylated inside mycobacteria but yet not reported the involvement of any specific mycobacterial kinase. We randomly selected some of the STPKs to look for the degree of phosphorylation of EspJ in *in vitro,* and made an observation that this protein is phosphorylated irrespective of the kinases used. Hence, we extended the studies using PknG which showed sustained and considerable effects on EspJ.

### Phosphorylation of EspJ by Ser/Thr protein kinase and mass spectrometric analysis

**Figure 2:**
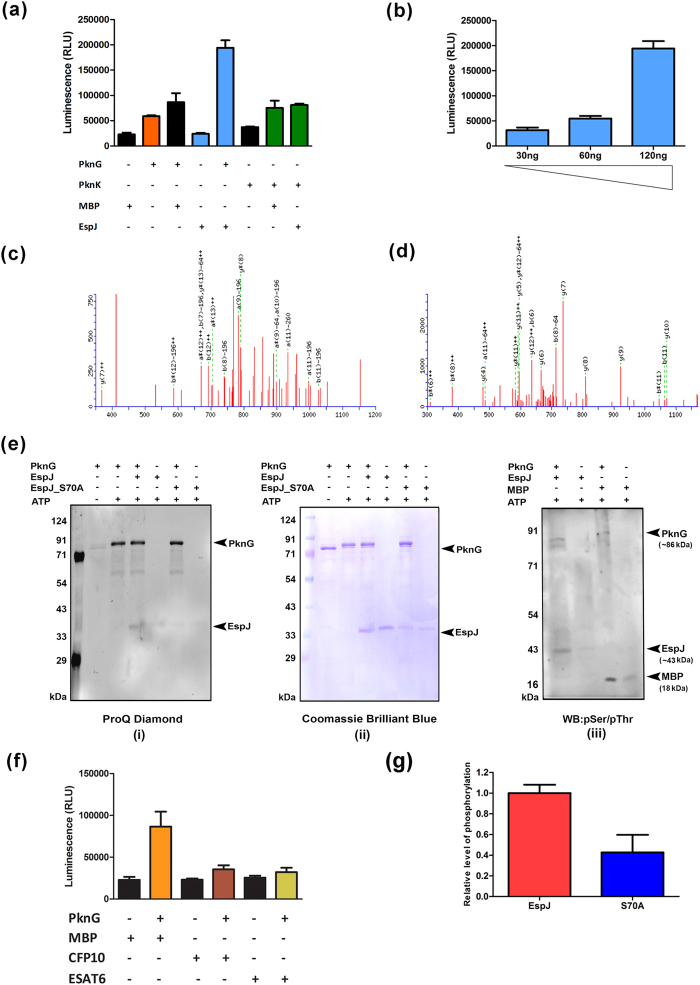
*In vitro* phosphorylation of EspJ protein (**a**) Kinase assay of STPK (PknG/PknK) with recombinant EspJ using kinase assay. MBP was used as universal substrate (**b**) PknG Kinase assay was done using varying concentrations of EspJ protein to show the linearity of dose dependent substrate specificity. (**c**) MS/MS (MS2) fragmentation of the modified peptide TA**pS**NMNAAADVYAK obtained by sequencing at MS2 level. The precursor mass of phospho-peptide at MS1 level (before sequencing / fragmentation) was 801.6 Da, which was 79 Da heavier as compared to previously obtained non-phosphorylated peptide i.e. 721.76 Da. (**d**) MS/MS fragmentation of un-phosphorylated peptide TASNMNAAADVYAK at MS2 level. Un-phosphorylated peptide didn’t show any phosphorylation showing the genuineness of phosphorylated residue by kinase reaction. (**e**) Relative level of phosphorylation of EspJ and phosphoablative mutant EspJ_S70A with PknG, quantified by ProQ diamond staining (left panel), and same blot was stained with coomassie stain to show the loading (middle panel). Since it was difficult to show the phosphorylation of MBP due to very low molecular weight (18.4 kDa), along with PknG (>80.0 kDa), the phosphorylations were detected by pS/T antibody on a gradient gel (right panel). (**f**) Kinase assay of STPK (PknG) with other RD1 encoded recombinant proteins CFP-10 and ESAT-6 and (**g**) Quantitative comparison of kinase assay using EspJ and phosphoablative mutant.

To substantiate bioinformatics predictions, recombinant EspJ was phosphorylated with PknG and with PknK *in vitro,* and was confirmed by kinase assay. Increased levels of phosphorylation were observed in EspJ as compared to MBP, which is used as a universal substrate (Fig. 2a,b). Phosphorylated EspJ was further resolved by 2DE and analyzed by mass spectrometry to confirm and identify the phosphorylation sites. The data reported phosphorylated residue at Ser^70^ position also by LC/MS/MS analyses of protein as compared to un-phosphorylated protein (Fig. 2c,d). The phosphorylation events were further ascertained by ProQ diamond staining (Fig. 2e) and by immunoblotting with pS/T antibody (Fig. 2e).The mutant protein was expressed and purified by Ni-NTA column. The kinase assay using the protein as substrate observed two fold reductions in the level of phosphorylation with PknG as compared to its wild type counterpart (Fig. 2f). In addition to EspJ, we have also used two other RD1 encoded secretory proteins CFP-10 and ESAT-6, to look whether these proteins are also getting phosphorylated by PknG or not. Kinase assay didn’t demonstrate a significant level of phosphorylation in these proteins (Fig. 2g). This experiment conclusively reported that among the secretory proteins of RD1 region, only EspJ is significantly phosphorylated.

### Comparative analysis of EspJ and its phosphoablative mutants in mycobacterial growth

**Figure 3:**
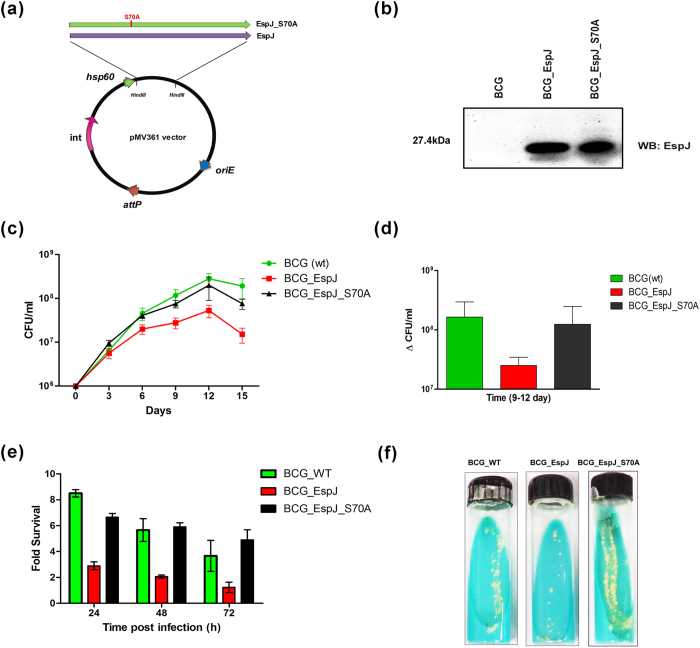
Role of EspJ protein in growth and in the survival of mycobacteria (**a**) Diagrammatical representation of cloning of *espJ* and its phosphoablative mutant alleles into mycobacterial integrative vector pMV361 (**b**) Western blotting with EspJ antiserum to confirm expression of EspJ and phosphoablative mutant proteins in recombinant BCG (**c**) Growth of recombinant *M. bovis* BCG containing vector alone (BCG), EspJ (BCG_*espJ*) and BCG_EspJ_S70A was recorded by CFU as mentioned in experimental procedure. (**d**) Bar diagram shows an increment in growth by recombinant BCG between 9-12 days and (**e**) Intracellular survival of recombinant *M. bovis* BCG in macrophages. THP-1 cells were infected with BCG, BCG_EspJ and BCG_EspJ_S70A as described in the experimental procedure. Aliquots of infected cells were lysed with 0.025% SDS at indicated times, and serial dilutions were plated on 7H10 agar plates containing kanamycin. Recovered CFUs were enumerated after the incubation for 20 days at 37 °C. Numbers of intracellular bacteria are shown in fold numbers detected at t = 24 h, 48 h and 72 h. Data are shown as means ± SD of triplicate experiments in three technical replicates. Similar results were obtained in three independent experiments. (**f**) Growth of mycobacteria on LJ slants, recovered from macrophages after 72 h, as discussed in (**e**).

To explore the role of EspJ protein, the gene was transferred into surrogate strain; *M. bovis* BCG. The growth of mycobacteria was monitored either by counting the CFU on MB7H10 medium plates or by MGIT 960 system. Temporal growth curve (growth unit verses time) as well as increment in growth units for BCG, containing EspJ and its mutants (Fig. 3a,b) were analyzed for the fixed period (9-12 day) of time. The results revealed the slow growth of recombinant BCG, which has a copy of *espJ* as compared to BCG, containing vector alone. This finding indicated towards the partial involvement of EspJ protein in slowing down the growth of mycobacteria. On the basis of this information and further to corroborate the effect of phosphorylation on EspJ, we generated phosphoablative mutants by site directed mutagenesis and transferred all these alleles into *M. bovis* BCG. Recombinant BCG expressing mutant allele EspJ_S70A restored the multiplication as compared to BCG over-expressing wild-type EspJ (Fig. 3c,d). The rBCG expressing other mutant alleles were also studied extensively, but didn’t show apparent restoration in growth defect (data not shown).

### Phagocytosis and intracellular survival of recombinant BCG expressing EspJ and phosphoablative mutants

To study the role of phosphorylation of EspJ in survival of mycobacteria, THP-1 cells were infected with BCG at MOI of 1:10 as described in experimental procedure. Phagocytosis was allowed to occur for 4 h. Cells were washed, and the remaining extracellular bacilli were killed by Amikacin treatment. Cells were then washed and lysed to release the intracellular bacilli. The suspension was then plated on MB7H10 plates supplemented with 10% OADC and the colony forming units (CFUs) were determined to enumerate the intracellular survival of recombinant BCG. Results showed that infection of macrophages with rBCG expressing phosphoablative mutants at Ser^70^ position significantly (p < 0.005) increases the survival of *M. bovis* BCG inside macrophages as compared to rBCG expressing wild type allele (Fig. 3e,f). Other mutants didn’t show any notable difference. The data also underlined that mutation at Ser^70^ position is responsible for increasing the growth of mycobacteria, is also important for persistent survival of mycobacteria inside the macrophages.

### Relative response of MTB containing wild type and phosphoablative mutant of *espJ*

**Figure 4:**
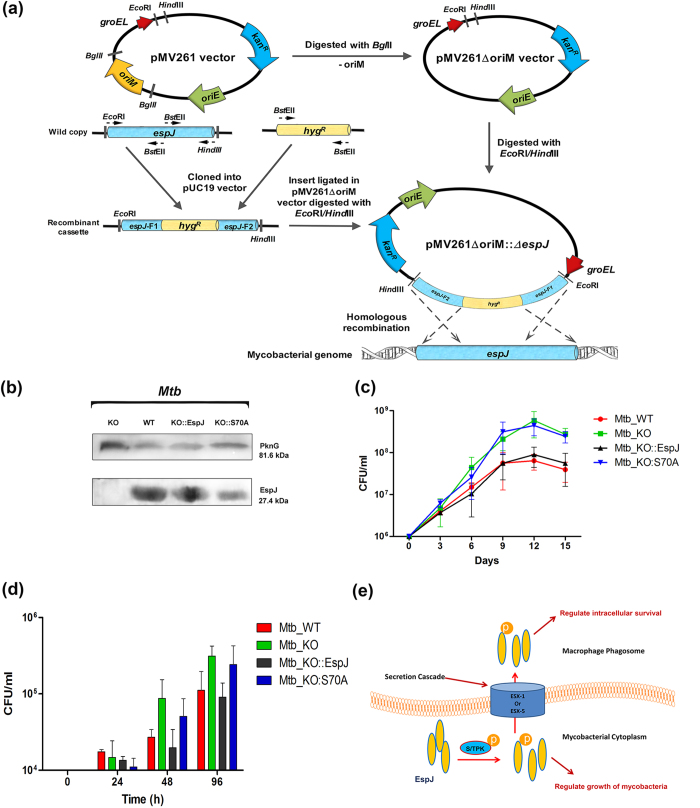
Growth kinetics of knock-out MTB Ra strain (**a**) Strategy and diagrammatical representation for the generation of knock-out (KO) construct of *Rv3878*. The *hyg* gene was inserted into *Rv3878* gene ORF to make it non-functional. The disrupted gene construct has been cloned in oriM^-^ pMV261 vector. (**b**) Western blot analysis of MTB KO, wild-type (WT), KO complemented with *espJ* (KO::EspJ) and KO complemented with *espJ_*S70A (KO::S70A) lysates. (**c**) Growths of MTB KO, WT, KO::EspJ and KO::S70A were monitored by CFU (**d**) Intracellular growth kinetics of MTB KO, WT, KO::EspJ and KO::S70A were recorded by CFU. Data are shown as means ± SD of triplicate experiments in three technical replicates. (**e**) The cartoon illustrates the role of RD1 encoded protein EspJ in mycobacterial growth. After being phosphorylated by PknG (S/T kinase) the EspJ regulates *in vitro* growth. It also gets secreted during infection and modulates the host protein for prolong intracellular survival.

We further ascertained the role of EspJ phosphorylation by generating MTB Ra knock-out strain (Fig. 4a,b). The growth kinetics of both knock-out and its complemented strains (wild type *espJ* as well as phosphoablative allele *espJ_S70A*) demonstrate involvement of this protein in growth of mycobacteria (Fig. 4c). We used these bacteria to study the intracellular survival inside THP-1 cells. The patterns of growth (Fig. 4d) were very similar to that shown in Fig. 4c. The *espJ* knock-out MTB multiplied more efficiently as compared to wild type strain. Since, EspJ has been reported to utilize Esx secretion system; we hypothesized that the events in intracellular survival may be due to transportation of phosphorylated protein in the macrophage (Fig. 4e).

### Phylogenetic analysis of EspJ orthologs in mycobacterial genus

**Figure 5:**
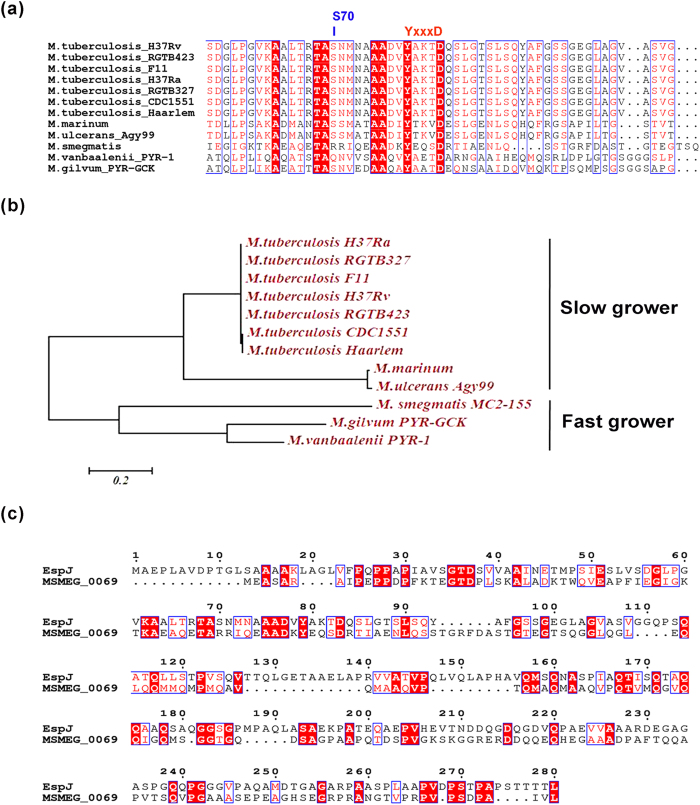
Closely related orthologs of EspJ protein found in slow grower mycobacterial strains. (**a**) Alignment of mycobacterial protein EspJ orthologs. Sequences of EspJ proteins retrieved from Tb database (http://www.tbdb.org/) were aligned using MUSCLE^29^. The program ESPript was used to generate figures of aligned sequences^32^. The Ser^70^ site mostly present in slow grower mycobacteria is shown with the arrow. (**b**) Phylogenetic tree of mycobacteria based on protein sequences of EspJ orthologs. Alignment of Protein sequence of closely related orthologs of EspJ was done by MUSCLE. The phylogenetic tree was constructed by the neighbor-joining method, using Jones-Taylor-Thornton substitution model using MEGA version 6^30^. (**c**) Alignment of EspJ and its ortholog MSMEG_0069 showing similarity of EspJ with translational initiation factor. Alignment was done as described in (**a**).

Phylogenetic study among closely related orthologs also reveals the presence of EspJ protein mostly in slow grower mycobacterial strains. Multiple sequence alignment using MUSCLE, demonstrated conservation of phosphorylation sites in a wide range of mycobacterial strains. Interestingly, Ser^70^ residue is also found to be important in slow grower mycobacteria as it is found to be substituted with other amino acids in fast growers (Fig. 5a,b).

## Discussion

With the advent of STPKs in mycobacteria it has been well documented that phosphorylation of proteins involved in determining the virulence, effects growth and later the pathogenicity of mycobacteria^15^,^16^. Alongside, it has also been reported that mycobacterial STPKs are expressed differentially in pathogenic and in non-pathogenic strains^9^. These findings indicate towards an existing relationship between mycobacterial STPKs and virulence factors which determine their differential response in diverse strains.

Deletion of certain regions of chromosome occurs during evolution of non-pathogenic mycobacteria from pathogenic counterpart *M. bovis*. RD1, a 9.5-kb DNA segment is deleted in all the BCG sub-strains^17^. Concurrently, Ra has also been evolved from its parental strain. Genomic analysis of Ra reveals deletion of chromosome region from Rv genome similar to events happened for attenuation of BCG^10^. Interestingly, in Ra; RD1 region which encodes several virulence factors^18^ remains intact. These events may infer a differential behavior of this protein in pathogenic and in non- pathogenic strains. It is established that post-translational modification is a central mechanism in modulating a protein for its differential activity. Based on these evidences we hypothesize that RD1 encoded protein may have undergone differential phosphorylation during the process. In this study, we made efforts to establish relationship between STPKs and RD1 encoded proteins and further its differential response in pathogenic and in non-pathogenic strains.

In order to understand the role of MTB STPKs signaling pathways in mycobacterial physiology, several efforts have been made to identify the substrates. It has previously been shown that proteins with the fork head-associated (FHA) domain get phosphorylated by STPKs of MTB and hence protein containing this motif has been identified as a substrate^19^,^20^,^21^. Similarly the RXS/T and RXXS/T motifs have been identified in Rv0019c and FtsZ proteins, which are reported to be phosphorylated by PknA^14^. Using *in silico* approach we have screened RD1 encoded proteins for the presence of such putative motifs. Besides that, web based tools like Kinasephos 2.0, Disphos 1.3, Netphos 2.0 and NetPhosBac1 have also been used to predict phosphorylation potential of all RD1 encoded proteins (Table 2). All these bioinformatics studies suggest presence of such motifs in RD1 encoded proteins including EspJ. At the outset, we looked for transcription of *espJ* and expression of the protein in Rv and in Ra. The results indicated that this protein is expressed in the late stage of mycobacterial growth and its phosphorylation is variable in virulent and in avirulent mycobacteria (Fig. 1).

The EspJ which was picked up among the RD1 encoded proteins through bioinformatics analysis, was further confirmed by *in vitro* kinase assay with PknG (one of the MTB STPKs). Although we could see the partial phosphorylation of EspJ with other STPKs (Fig. 2a), nevertheless the purpose of addressing this mechanism was to look the differential nature of EspJ in phosphorylated and in unphosphorylated forms, irrespective of the kinase used. Although the promiscuity of PknG cannot be claimed over other STPKs in mycobacteria, the rationale behind selecting PknG, is that it acts as a virulence factor and its differential expression occurs in pathogenic and in non-pathogenic strains of MTB^9^. Apart from that, PknG regulates mycobacterial physiology via phosphorylation of a wide range of substrates. It also regulates glutamate metabolism via phosphorylation of GarA^22^. Kinase assay suggests possible phosphorylation of EspJ protein encoded by *Rv3878* gene (Fig. 2a,b). Further, in order to identify phosphorylation site(s), we used mass spectrometry, which is generally used to identify phosphorylation site in phospho-proteins^23^.

Mass spectrometry data reveal Ser^70^ as a major phosphorylation site in EspJ protein (Fig. 2c,d). To authenticate the phosphorylation site, phosphoablative mutant for Ser^70^ was generated by site directed mutagenesis. The *in vitro* kinase assay using phosphoablative mutant as a substrate showed the abrogation of phosphorylation of EspJ protein, which confirmed the involvement of Ser^70^ residue (Fig. 2e,f).

Phosphorylation may occur at several positions in a protein. We used the bioinformatics tool to identify several putative phosphorylation sites in EspJ protein, which are characteristic features of phospho-residue of MTB STPK, like presence of RXXS/T motifs. We made mutants, at all such positions, but didn’t get significant difference in the phosphorylation level by *in vitro* kinase assay with PknG (data not shown).

We further predicted the possible role of EspJ in relation to mycobacterial physiology. Although, most of the orthologs of EspJ are uncharacterized, its closest ortholog MSMEG_0069 (*M. smegmatis*) has been annotated as translation initiation factor IF-2. Alignment of MTB EspJ (Rv3878) with MSMEG_0069 demonstrates large similarity between these two proteins (Fig. 5c). Earlier studies suggest involvement of phosphorylation as a mechanism to modulate translational event. In prokaryotes like in streptomycetes, ribosome-associated STPKs, phosphorylate 11 proteins which result in 30% loss of ribosomal activity^24^. Apart from that, activity of elongation factor-Tu involved in protein synthesis of MTB, is largely dependent on its phosphorylation^25^. Since, EspJ protein has been annotated as a regulatory protein in *M. smegmatis,* and is associated with the category of cell wall and cell processes^12^, we articulated that EspJ might participate in the growth and in survival of mycobacteria. To analyze whether presence of this protein is specific to slow grower mycobacteria; all available mycobacterial genome sequences were analyzed for the presence of a locus encoding EspJ protein. As evidenced from phylogenetic tree, EspJ is mostly present in slow grower mycobacteria (Fig. 5a,b). Moreover, substitution of Ser^70^ site with phosphoablative alleles in slow grower mycobacteria intrigues us to see the biological relevance of this site in modulation of growth behavior of mycobacteria. The EspJ of mycobacteria is associated in virulence, while the similar kind of protein in *E. coli* has been predicted to be involved in secretion system and controlling the Src kinase activity^26^. We analyzed the protein sequence of these two proteins and inferred that although they share the same name but are entirely heterologous.

To corroborate the biological effect of this protein we used *M. bovis* BCG as a surrogate model strain which contains all the STPKs but lacks EspJ due to deletion of RD1 region. Expression of PknG in *M. bovis* BCG was confirmed prior to use it as a surrogate strain (Fig. 1c). Recombinant BCG strains were generated by transferring *espJ* alleles downstream of the *hsp*60 promoter through integrative vector pMV361. Interestingly, we observed the slow growth of recombinant BCG as compared to wild type. This study along with phosphorylation event encouraged us to further investigate the effect of EspJ phosphorylation, on the growth of mycobacteria. When we transferred phosphoablative mutant allele EspJ_S70A in *M. bovis* BCG, we observed a considerable amount of increase in the growth of rBCG as compared to rBCG having wild type EspJ allele. Abolition of the growth defect after transferring EspJ_S70A allele in *M. bovis* BCG suggests involvement of this residue in phosphorylation by STPKs. Interestingly this residue is similar to phosphorylated residue found in FtsZ protein regulating cell division of mycobacteria and is part of Rxx(S/T) motif found in most of the phosphorylated residues in MTB^14^.

The role of EspJ phosphorylation in mycobacterial growth has also been established by gene knockout strategy in MTB (Fig. 4). Overall, these findings for the first time demonstrate the involvement of phosphorylation as one of the mechanisms through which STPK associate with EspJ and orchestrate the growth of mycobacteria.

Since, a large proportion of EspJ is secreted into the culture medium and in macrophages (Fig. 1e,f); we detected its comparative phosphorylation level between Rv and Ra in a log phase culture filtrate to delineate the *in vivo* phosphorylation of EspJ by STPKs. As expected, we found a higher level of phosphorylation in pathogenic strain compared to non pathogenic strain (Fig. 1d). We checked whether this phenomenon is not due to necrosis of the bacilli, we confirmed, using the hsp70 antibody. Since, hsp70 is not a secretory protein; we were unable to detect the bands on the blot. In agreement to our findings, a recent report on PE/PPE protein implicated the differential phosphorylation between Rv and Ra strains, which helped in determining the pathogenic phenotype^27^. Differential phosphorylation of EspJ, encoding a virulence factor suggests that loss of phosphorylation may be a mechanism adopted by non-pathogenic mycobacteria during evolution from pathogenic mycobacteria.

In summary, the phosphorylation of RD-1 encoded protein is unique and may be critical for growth and survival of mycobacteria. One of the proteins encoded by this region; EspJ undergoes phosphorylation by STPK as evidenced by LC/MS/MS analyses. Proteins of pathogenic (Rv) and nonpathogenic (Ra) mycobacteria can be distinguished based on the variable levels of phosphorylation of EspJ. Study with a surrogate strain, *M. bovis* BCG; where STPKs are present and EspJ is deleted, demonstrated that in order to sustain the growth of mycobacteria, EspJ gets phosphorylated. This was further confirmed by complementing the phosphoablative mutant of EspJ in KO strain of MTB.

## Methods

### Mycobacteria cultivation and growth

Mycobacterial strains (Table 3) were cultured in Middlebrook 7H9 medium (Difco) supplemented with Albumin Dextrose Catalase (BD). A suspension of bacilli was prepared in 10 ml Middlebrook 7H9 broth by repeated vortexing log phase culture of mycobacteria. 0.5 ml of the diluted (10^–3^) culture was transferred to BBL MGIT tube and incubated at 37 °C, and monitored for the change in fluorescence. The BACTEC MGIT 960 system monitors fluorescence (in Growth units) every hour. The growth obtained was recorded about every 24 h during the first 120 h. The prepared dilutions were also plated on MB7H10 agar and incubated at 37 °C to detect CFU and to eliminate the possibility of any other bacterial contaminations.

### Plasmid construction, mutagenesis and protein purification

The ORFs of *pknG* and RD1 encoded *espJ* were amplified from MTB Rv genomic DNA using flanking *Hind*III anchored primers (Table 4). Both the genes were subcloned into pTriEx-4, expression vector (Novagen) and were used to transform *E. coli* BL21 (DE3). *E. coli* strains DH5α and BL21 (DE3) were cultured in Luria–Bertani medium. Over-expressions of His-tag recombinant proteins were done using 0.2 mM IPTG concentration at 18 °C for overnight and purification was done with Ni-NTA column chromatography. The protein showed a relative molecular mass of 43.0 kDa in *E coli*, while in mycobacteria it is expressed as 27.4 kDa monomeric protein. In addition, *espJ* was also sub cloned downstream of the hsp60 promoter into pMV361 vector. This vector contains an *E. coli* origin of replication (oriE), the *attP* and *int* genes of mycobacteriophage L5 (for integration in the mycobacterial chromosome) and a kanamycin resistance marker (Kan^r^). The resulting plasmid was used for complementation in *M. bovis* BCG to explore the role of *espJ* and generation of phosphoablative mutants by site directed mutagenesis at T68V, S70A, S85A and T144V positions by PCR using overlapping primers (Table 4). The veracity of all the clones was checked by DNA sequencing. Confirmed clones having wild type as well as mutated alleles were electroporated into *M. bovis* BCG^28^. Recombinant expressions of these proteins in BCG were ascertained by western blotting using EspJ antiserum.

### Antibodies

Polyclonal antisera for PknG, GarA and EspJ were raised in the rabbits in the animal facility of the institute. All the animal experiments were performed in accordance with the approved guidelines of CSIR- Central Drug Research Institute, Lucknow. Albino rabbits (1.5 kg) were obtained from the animal colony of the institute. The animals were maintained in an animal house accredited by the National Accreditation Board for Testing and Calibration Laboratories guidelines controlled by the Committee for the Purpose of Control and Supervision of Experiments on Animals (CPCSEA). CSIR-Central Drug Research Institute constituted ethics committee for use of laboratory animals has approved all the protocols for the study. The animals were maintained in standard conditions of temperature and humidity (temperature 22 ± 2 °C; humidity 45–55%; light intensity 300–400 lx) and given proper pellet diet and water ad libitum. Antibodies for hsp70 (Sigma) and pSer/Thr (Qiagen) were procured commercially.

### Bioinformatics analysis

Phosphorylation potential as well as putative phosphorylation sites in RD1 encoded proteins were predicted by publicly available online analysis tool (Table 2); Netphos 2.0 (http://www.cbs.dtu.dk/services/NetPhos/), NetPhosBac1 (http://www.cbs.dtu.dk/services/NetPhosBac-1.0/), DISHPHOS 1.3 (http://www.dabi.temple.edu/disphos/pred.html) and, Kinasephos 2.0 (http://kinasephos2.mbc.nctu.edu.tw/). Alignment of protein sequence of closely related orthologs of EspJ was done by MUSCLE^29^. Phylogenetic and molecular evolutionary analyses were conducted using MEGA version 6^30^. The phylogenetic tree was constructed by the neighbor-joining method, using Jones-Taylor-Thornton substitution model.

### *In vitro* kinase assay

To validate the MS-MS data for phosphorylation sites; quantitative and qualitative *in vitro* kinase assays were performed with either using ProQ Diamond (Invitrogen) or ADP-Glo™ Kinase Assay (Promega) according to manufacturer’s instruction. For ADP-Glo assay, PknG (25 ng) was used as a kinase and incubated either with EspJ or with the mutants (100 ng) in buffer containing 40 mM Tris (pH 7.5), 2 mM MnCl_2_, 20 mM MgCl_2_, 2 mM DTT, 0.5 mg/ml BSA, and 0.1 mM ATP for 1 h at 26 °C. The assay was also standardized using different concentrations of substrate (100, 50 and 25 ng).

### 2D gel electrophoresis and mass spectrometry analyses

Purified recombinant protein EspJ was subjected to *in vitro* kinase assay as described above, and 2DE was done to resolve these two proteins, using a standard protocol. Phosphorylated as well as un-phosphorylated EspJ spots from two different gels were excised and digested with Trypsin and processed further for LC/MS/MS analyses by HCT Ultra PTM Discovery System (ETD II-Bruker Daltonics) with 1100 series HPLC (Agilent) to identify phosphorylated sites. Briefly, the protein spots were excised from the coomassie stained gel, diced into small pieces in microcentrifuge tubes. The gel pieces were destained by washing them twice with 200 μl of 25 mM ammonium bicarbonate (ABC, Sigma).The gel pieces were dehydrated thrice with 50 μl of a 2:1 mixture of acetonitrile (LC-MS CHROMASOLV, Sigma) and 50 mM ABC for 5 minutes and twice with 25 mM ABC for 2 minutes. The proteins were reduced with 10 mM DTT in 100 mM ABC at 60 °C for 1 hr, washed with 25 mM ABC and alkylated with 100 mM iodoacetamide at room temperature for 20 minutes to alkylate the cysteine residues. Gel slices were dehydrated twice with two washes of acetonitrile followed by a final wash with 25 mM ABC. The gel pieces were dried in speed vac for 15 minutes and rehydrated with 30 μl of 20 μg/ml solution of trypsin (Proteomics grade, Sigma) overnight at 37 °C. The supernatant was acidified with 0.1% trifluoroacetic acid (TFA, Sigma) and a 1:1 (v/v) mixture of the sample and α-Cyano-4-hydroxycinnamic acid (CHCA) was spotted on the MALDI plate prior to analysis. The mgf file generated after the sample run was used for MTB UniProt database search using a Mascot software package (Matrix Science London). The peptide precursor mass tolerance was set to 1.2 Da, and fragment ion mass tolerance was set to 0.6 Da. Carbamidomethylation on cysteine residues was used as fixed modification and oxidation of methionine and phosphorylations of serine, threonine, and tyrosine were used as variable modifications. Phosphopeptides were detected in phosphorylated sample.

### THP-1 macrophage cells infection with recombinant mycobacteria

The macrophage cell line THP-1 was cultured at 37 °C and 5% CO_2_ in RPMI-1640 medium (2 mM L-glutamine,10 mM HEPES, 1 mM sodium pyruvate, 4.5 g/L glucose and 1.5 g/L sodium bicarbonate), supplemented with 10% heat inactivated fetal calf serum (FCS). The cells were differentiated into macrophage-like cells by treatment with phorbol myristate acetate (PMA) before infection. The cells having a density of 5 × 10^5^ cells/ml were seeded in tissue culture plates. The single cell suspension of log-phase mycobacteria was prepared by vortexing in presence of sterile glass beads and passing through a 26 gauge needle and was used for macrophage infection (MOI = 1:10) as described^31^. After infection, cells were washed with incomplete RPMI-1640 medium thrice and extra-cellular bacteria were removed by killing with 100 μg/ml amikacin. The macrophages were lysed with 0.05% SDS at different time points (0, 24 h, 72 h and 96 h) to recover the bacteria and enumerated by plating appropriate dilutions on MB7H10 plates containing 10% OADC (Oleic acid albumin dextrose complex) and kanamycin (25 μg/ml). After recovery, CFUs were counted at the different time points.

### Preparation of cell lysate and culture supernatant proteins of mycobacteria

Mid-log phase cultures were harvested by centrifugation at 2500*g* for 15 minutes. Cell pellets were washed twice with PBS. Cell pellets were re-suspended in protein extraction buffer (50 mM Tris, 100 mM NaCl, protease inhibitor cocktail) and disrupted by bead beating. The protein loading buffer was added to cell lysates, and samples were boiled for 15 min before loading onto SDS-PAG. Since albumin confounded analysis of secreted bacterial proteins we have used Sauton medium supplemented with 0.05% Tween 80 instead of 7H9 medium. To analyze secretory proteins by SDS-PAGE, mid-log phase mycobacterial strains were resuspended in fresh medium and cultured overnight at 37 °C. Culture supernatants obtained after centrifugation at 2500*g* for 15 minutes and sterilized by double filtration through 0.2-μm filters were treated with trichloroacetic acid (TCA, w/v) (Sigma, St. Louis, USA), to finally obtain a final concentration of 10% (w/v). The resulting mixture was kept at −20 °C for 5 h and centrifuged at 18,000 g for 30 min at 4 °C. Pellets thus obtained were washed thrice with pre-chilled acetone (Sigma, St. Louis, USA) and re-suspended in Laemelli buffer for western analysis.

### RNA isolation and quantitative RT-PCR (qRT-PCR)

RNA was isolated from log and stationary phase cultures of mycobacterial strains Rv and Ra, using the RNeasy Mini kit (Qiagen) according to manufacturer’s instructions. DNase treated RNA was used for cDNA synthesis using first-strand cDNA synthesis kit (Fermentas) and random hexamer primers. Quantitative real time PCR was performed in 96 well plate on Applied Biosystems StepOne™ Real-Time PCR Systems using Power SYBR®Green PCR master mix (Applied biosystem) with 5 pM primers (Table 4). The calculated cycle threshold (C_t_) of the test gene was normalized to the C_t_ of the internal control (*sigA and 16S*) genes, before calculating the fold change in the test samples.

## Additional Information

**How to cite this article**: Singh, P. K. *et al.* RD-1 encoded EspJ protein gets phosphorylated prior to affect the growth and intracellular survival of mycobacteria. *Sci. Rep.* **5**, 12717; doi: 10.1038/srep12717 (2015).

## Figures and Tables

**Table 1:** Co-inheritance of MTB STPKs and RD1 encoded proteins.

**Mycobacteria Sp**	**RD-1**	**MTB STPK**	**Multiplication in Host**	**Disease**
MTB H37Rv	Yes	Yes	Yes	Yes
BCG	No	Yes	Yes	No
MS	Non-Homologous	No (except A & B)	No	No
MTB H37Ra	Yes	Yes	Yes	No

**Table 2:** Prediction of phosphorylation potential of RD1 encoded proteins by different bioinformatic tools.

**RD1 encoded protein**	**Total no. of amino acid**	**Number of potential phosphorylation sites predicted**							
		**Kinasephos 2.0 (score > 0.8)**		**DISPHOS 1.3 (score > 0.5)**		**NETPHOS 2.0 (score > 0.5)**		**NETPHOS BAC1 (score > 0.5)**	
		**Serine**	**Threonine**	**Serine**	**Threonine**	**Serine**	**Threonine**	**Serine**	**Threonine**
EccCb1	591	25	1	15	9	15	6	6	2
PE35	99	9	0	2	0	3	0	0	0
PPE68	368	26	1	15	7	6	6	4	0
EsxB	100	6	0	3	1	1	0	1	0
EsxA	95	8	0	4	0	3	1	11	2
EspI	666	38	2	22	20	21	11	11	1
EccD1	511	16	2	6	13	9	11	11	1
EspJ	280	26	0	18	5	7	2	2	1
EspK	729	36	4	22	40	22	21	9	1

**Table 3:** Strains and plasmids used in this study.

**Strains**	**Description**	**Source**
*E. coli* DH5a	Standard gene cloning strain	NEB
*E. coli BL21* (DE3)	Gene expression strain	NEB
*M. tuberculosis* H37Rv	Virulent laboratory strain	ATCC25618
*M. tuberculosis* H37Ra	Avirulent strain	ATCC25177
*M. smegmatis* mc2 155	*ept-1*	ATCC700084
*M. bovis* BCG	Attenuated strain, lacking RD1 region	ATCC35734 (TMC 1011 BCG Pasteur)
BCG_S68A	rBCG expressing *espJ_*S68A allele	This study
BCG_S70A	rBCG expressing *espJ*_S70A allele	This study
BCG_S85A	rBCG expressing *espJ*_S85A allele	This study
BCG_T144V	rBCG expressing *espJ*_T144V allele	This study
THP1	Macrophage cell line	ATCC
Plasmids		
pTriEx4	Expression vector, amp^r^, 6xHis-tag	Novagen
pTriEx4::*pknG*	Expression vector having *pknG*	This study
pTriEx4::*espJ*	Expression vector having *espJ*	This study
pMV361	*E. coli*-Mycobacteria shuttle vector, groEL2 (hsp60) promoter, Kan^r^, OriM	This study
pMV361::*espJ*	pMV361 containing wild type *espJ*	This study
pMV361::*espJ*_T68V	pMV361 containing *espJ*_S68A	This study
pMV361::*espJ*_S70A	pMV361 containing *espJ*_S70A	This study
pMV361::*espJ*_S85A	pMV361 containing *espJ*_S85A	This study
pMV361::*espJ*_T144V	pMV361 containing *espJ*_T144V	This study
pMV261*_∆espJ*	pMV261 containing *espJ_hyg* allele, OriM^-^	This study

**Table 4:** List of primers used in this study.

**A. Site directed mutagenesis primers**	**Sequences (5′ to 3′)**
1. EspJ_T68VR	GTTCATGTTGGATGCTACTCGAGTCAGGGCGGCTTTCA
2. EspJ_T68VF	TGAAAGCCGCCCTGACTCGAGTAGCATCCAACATGAAC
3. EspJ_S70AF	CCCTGACTCGAACAGCAGCCAACATGAACGCG
4. EspJ_S70AR	CGCGTTCATGTTGGCTGCTGTTCGAGTCAGGG
5. EspJ_S85AF	GCGAAGACCGATCAGGCACTGGGAACCAGTT
6. EspJ_S85AR	AACTGGTTCCCAGTGCCTGATCGGTCTTCGC
7. EspJ_T144VF	CCCGTGTTGTTGCGGTGGTGCCGCAACTCG
8. EspJ_T144VR	CGAGTTGCGGCACCACCGCAACAACACGGG
B. Gene cloning primers	
1. EspJ_F	AAGCTTATGGCTGAACCGTTGGCC
2. EspJ_R	AAGCTTCTACAACGTTGTGGTTGTTG
3. PknG_F	CCCAAGCTTATGGCCAAAGCGTCAGAGAC
4. PknG_R	CCCAAGCTTTTAGAACGTGCTGGTGGGCC
C. Real time primers	
1. EspJ_RT_F	GTGAAAGCCGCCCTGACTC
2. EspJ_RT_R	ACGAGCCGAATGCATACTGG
3. SigA_RT_F	CGCCGATGACGACGAGGAG
4. SigA_RT_R	CTTGCCGATCTGTTTGAGGTAGG
